# The Effect of Soluble Fiber Dextrin on Subjective and Physiological Markers of Appetite: A Randomized Trial

**DOI:** 10.3390/nu12113341

**Published:** 2020-10-30

**Authors:** Christine H. Emilien, Walter H. Hsu, James H. Hollis

**Affiliations:** 1 Department of Food Science and Human Nutrition, Iowa State University, Ames, IA 50011, USA; Christine.Emilien@gmail.com; 2 Department of Biomedical Sciences, Iowa State University, Ames, IA 50011, USA; whsu@iastate.edu

**Keywords:** appetite, fiber, satiety, breath hydrogen, resistant starch

## Abstract

Obesity is a leading public health problem throughout the world. The development of foods that increase satiety and reduce food may aid weight management. This study determined the effect of consuming soluble fiber dextrin (SFD) on appetite, appetitive hormones, breath hydrogen and food intake in adults. Forty-three participants completed this study. For each treatment, 50% of the SFD was provided in liquid form as part of breakfast and 50% in solid form as a morning snack. Appetite questionnaires, blood and breath samples were collected immediately before breakfast and at regular intervals during the test session. The participants consumed an ad libitum lunch meal, afternoon snack and dinner meal, and the amount eaten was recorded. Following dinner, participants left the laboratory but were required to keep a diet diary for the remainder of the day. Breath hydrogen concentration was significantly higher following the consumption of SFD compared to control (*p* < 0.05). There was no observed overall treatment effect of consuming SFD on GLP-1 (Glucagon-Like-Peptide-1), ghrelin, CCK-8 (Cholecystokinin) or PYY_3-36_ (Petptide YY) (*p* > 0.05). Moreover, consuming foods containing SFD had no effect on subjective appetite or food intake (*p* > 0.05). Consuming foods containing SFD increased breath hydrogen but did not influence food intake, appetite or appetitive hormones. However, the limitations of this study may have individually or collectively masked an effect of SFD on food intake and appetite.

## 1. Introduction

Throughout the world, the number of overweight and obese adults has risen markedly over the past few decades. This is of concern as these conditions are associated with increased risk of chronic diseases such as type 2 diabetes [1], cardiovascular disease [2], or cancer [3,4]. Consequently, reducing the number of overweight or obese individuals is a leading public health goal. One potential strategy to aid weight management is to develop foods that enhance satiety so that their regular consumption leads to a reduction in food intake and weight loss.

Fibers can be isolated from plant foods and added to other foods to provide physiological or metabolic benefits (e.g., reduction in post-prandial plasma glucose) and reduce the risk of chronic disease. One potential benefit of adding fiber to foods is increased satiety which may aid in weight management. However, the effect of fiber on satiety is currently unclear with studies providing divergent results [5]. The inconsistent effects of fiber on satiety is likely due to the large differences in the physical and chemical characteristics of different fibers [6]. For instance, fibers can differ in their fermentability, viscosity, solubility and hydration properties [7]. Consequently, the effects of individual fibers on satiety may not be predictable and requires testing.

Resistant starch is a type of fiber that could potentially be incorporated into a wide variety of food products to augment satiety [8]. Accumulating evidence from studies using rodent models indicate that replacing rapidly digestible starch with resistant starch reduces weight gain in obesity-resistant and obesity-prone rats [9,10]. As resistant starch provides fewer calories than starch, this may be due to an energy dilution effect [11]. However, several studies conducted in humans suggest that resistant starch may also aid weight management by enhancing satiety [12,13,14].

Soluble fiber dextrin (SFD) is a resistant dextrin derived from starch. Emerging evidence suggests that SFD may reduce appetite and food intake in the short-term. For instance, providing SFD in a beverage reduced food intake in humans [13]. Moreover, a recent study found that the consumption of SFD did not influence appetite in the first two hours after consumption but an effect on appetite was noted several hours after consumption [15]. A possible explanation for this delay in satiety is that it takes several hours for the SFD to reach the large intestine where it is likely fermented to produce short-chain fatty acids (SCFA) [16]. Recent studies using colonic cells report that SCFA trigger the release of the hormones GLP-1 ((Glucagon-Like-Peptide-1) and PYY_3-36_ (Peptide YY) [17,18] which are related to satiety [19]. Consequently, SFD may need to reach the large intestine before it exerts an effect on appetite.

The primary objective of this present study was to determine the effect of consuming different doses of SFD on food intake over a single day. The secondary objectives were to determine the effect of SFD on subjective appetite, satiety-related hormones and breath hydrogen as these may provide a mechanistic explanation for an effect on food intake. Additionally, it was determined if there was a dose-response effect and if there were any differences between the SFD derived from corn and tapioca starch. We hypothesized that participants consuming SFD would reduce their food intake over a single day due to increased satiety. In addition, we hypothesized that the increased satiety would be explained by increased breath hydrogen, higher CCK-8 (Cholecystokinin-8), GLP-1 and PYY_3-36_, and lower ghrelin. In addition, we hypothesized that the effect on appetite would be dose-dependent (increasing the SFD dose will have a larger effect on the outcome measures) and that there would be no difference between the sources of SFD (corn vs. tapioca).

## 2. Materials and Methods

### 2.1. Participants

Healthy adults aged 18–45 years with a body mass index (BMI) of 19.9–29.9 kg/m^2^ were recruited via a mass email sent to Iowa State University faculty, students and staff, and through flyers posted in the local community. This study extends a previous study that suggested that SFD may influence appetite several hours after consumption in individuals with these characteristics [15]. Individuals interested in the study were invited to a screening session where their height and weight were measured and they were asked to complete a questionnaire that posed questions about their general health and attitudes to food. Additionally, during the screening, participants were asked to taste test foods used in the study (SFD beverages, bars and foods used for ad libitum test meals) and to rate the palatability of each item on a scale from 1 (least palatable) to 9 (most palatable). Participants were excluded from the study if they: were outside the target age or BMI range, were not weight-stable (weight change of 3 kg or more in the past 3 months), did not regularly consume breakfast and afternoon snacks, had a presence or history of gastrointestinal disease or food intolerance, were a restrained eater (≥14 on the restraint section of the three-factor eating questionnaire) [20], did not find the test foods palatable (<5 on a 9-point scale), or were using medication that lists a side effect on appetite. All subjects gave their informed consent for inclusion before they participated in the study. The study was conducted in accordance with the Declaration of Helsinki, and the protocol was approved by the Institutional Review Board of Iowa State University (#14-020). The ClinicalTrials.gov Identifier is NCT04596969.

### 2.2. Protocol

This study used a double-blind, randomized, cross-over design. Data were collected at the Nutrition and Wellness Center at Iowa State University. All study foods were provided in unlabeled packages and the researchers did not know the identity of the products until after the statistical analysis had been completed. All participants reported to the laboratory on five separate occasions with at least one week between each test session. The participants were assigned to a treatment order using a random number generator by the study staff. Participants were instructed to avoid consuming alcohol or conducting strenuous activity in the 24 h prior to each test session. On the evening before each test session, participants were asked to consume a standardized evening meal that had been provided by the research team. The participants were asked to finish eating the meal by 9:00 p.m. and to refrain from consuming any further foods or beverages, except water, until reporting to the laboratory at 7:30 am the following morning. On reporting to the laboratory, the participant’s body weight was measured using clinical weighing scales (Detecto 758C, Cardinal Scale Manufacturing Company, Webb City, MO, USA). They were then taken to a quiet room where an indwelling catheter was inserted into their non-dominant arm by a registered nurse. The participant was allowed to acclimatize to the indwelling catheter for 30 min before a baseline blood draw and breath sample were taken. A baseline appetite questionnaire was also completed. The participant was then provided with a breakfast meal that provided 20% of their estimated daily energy requirements which they were required to eat in its entirety within 15 min. The breakfast included one of the five SFD test beverages. On completion of this meal, another blood draw and breath sample were collected and an appetite questionnaire was completed (*t* = 0). At 10:15 am (*t* = 120 min), one of five SFD test bars was served as a mid-morning snack which the participant was required to eat in its entirety within 5 min of serving. An ad libitum lunch meal in excess of what would reasonably be expected to be consumed was served at 12:30 pm (*t* = 240 min). Participants were instructed that they had 15 min to eat until comfortably full, after which, the meal was withdrawn. A mid-afternoon snack, in excess of what could reasonably be eaten, was provided at 3:00 pm (*t* = 390 min). Participants were instructed that they had 10 min to eat until comfortably full. After the final blood draw was taken (*t* = 600 min), the indwelling catheter was removed from the participant’s arm. An ad libitum evening meal was served and the participant was instructed to eat until comfortably full. Following this meal, the participant could leave the laboratory and they were asked to keep a diet diary to record all food and beverage intake for the remainder of the day. These diaries were analyzed to determine energy and macronutrient intake using Nutritionist Pro™ Diet Analysis Software (version 2.1.13; First DataBank, San Bruno, CA, USA). During the test day, participants were asked to complete twenty-four appetite questionnaires at regular intervals throughout the test day (*t* = 15, 30, 45, 60, 90, 120, 135, 150, 165, 180, 240, 255, 270, 285, 300, 360, 390, 405, 420, 435, 450, 480, 540 and 600 min after breakfast consumption). Breath samples were collected in two hour intervals (*t* = 120, 240, 360, 480 and 600 min after breakfast consumption). Additionally, blood samples were collected twelve times following the breakfast meal (*t* = 60, 120, 180, 240, 270, 300, 260, 290, 420, 480, 540 and 600 min after breakfast consumption).

### 2.3. Test Foods and Beverages

The SFD used in the present study was derived from two different sources: corn and tapioca. Both sources of SFD provide 50% fiber and 50% digestible carbohydrate; therefore, the two doses (20 and 40 g SFD) tested provided 10 and 20 g of fiber, respectively. Five treatments were used in this study: control (0 g SFD and 10 g maltodextrin), corn10 (20 g SFD to provide 10 g fiber), corn20 (40 g SFD to provide 20 g fiber), tapioca10 (20 g SFD to provide 10 g fiber) and tapioca20 (40 g SFD to provide 20 g fiber). For each treatment, half of the SFD dose was provided with breakfast and half as part of a mid-morning snack. The SFD was consumed as a milk-based beverage with breakfast and as an ingredient in a snack bar product at the mid-morning snack. The doses selected were based on previous research [15] and the technical considerations of including fiber in the test foods.

The standardized dinner meal consumed on the evening before the test session included chicken nuggets (Tyson Foods Inc., Springdale, AR, USA), barbeque sauce (Kraft Food Groups Inc., Northfield, IL, USA), mashed potatoes (Idahoan Foods, Idaho Falls, ID, USA), Great Value mixed vegetable (Wal-Mart Stores Inc., Bentonville, AR, USA), and chocolate chip cookies (Nabisco^®^, East Hanover, NJ, USA) for dessert. This meal provided 814 kcal calories and had a macronutrient profile of 15% protein, 46% carbohydrate and 39% fat.

For the breakfast meal, participants’ basal metabolic rate was estimated using validated equations [20]. This figure was multiplied by 1.3 to estimate total daily energy (TEE) requirements. Not including the test beverage, the breakfast meal provided 20% of the calculated TEE with a macronutrient profile of 13% protein, 61% carbohydrate and 26% fat. Foods included in the meal were hard-boiled egg (Crystal Farms, Minnetonka, MN, USA), pineapple chunks (Dole Food Company, Westlake Village, CA, USA), cinnamon raisin bagel (Bimbo Bakeries, Fort Worth, TX, USA) and salted butter (Land O Lakes^®^, Arden Hills, MN, USA).

Following the mid-morning snack bar, which provided the second half of the daily SFD dose, ad libitum meals were served for lunch, afternoon snack and dinner. The lunch meal consisted of pasta and tomato sauce (Barilla Group, Parma, Italy) with Kraft^®^ shredded parmesan cheese. The afternoon snack was Classic Lay’s^®^ potato chips (PepsiCo Inc., Purchase, NY, USA) and dinner was chicken fried rice (Kahiki^®^ Foods Inc., Colombus, OH, USA). All meals and snacks were served with an 8 oz bottle of Dasani^®^ water (The Coca-Cola Company, Atlanta, GA, USA). Participants were not allowed to eat or drink outside of the test foods and drinks provided.

### 2.4. Subjective Appetite and Food Intake

Participants completed a standard appetite questionnaire contained on a PalmPilot. The following questions were asked: How hungry do you feel right now? How full do you feel right now? What is your desire to eat right now? What is your prospective consumption right now? Responses were measured using a visual analogue scale anchored with opposing statements at each end (e.g., not hungry at all or as hungry as I have ever felt). Answers were captured and stored with a time and date stamp so compliance to the study protocol could be determined.

### 2.5. Hormones and Breath Hydrogen 

Blood was drawn into EDTA (Ethylenediaminetetraacetic acid)-coated vacutainers, mixed with a relevant preservative and centrifuged. The plasma was collected and stored at −80 °C until being assayed. Plasma samples were ethanol-extracted [21] before being analyzed for GLP-1, CCK-8 and PYY_3-36_ using established RIA (radioimmunoassay) procedures [22,23]. Ghrelin was also measured via radioimmunoassay but used unextracted blood samples. For all analyses, samples were run in duplicate and all samples from a given participant were analyzed within the same batch. All ^125^I-Tracers used were purchased from PerkinElmer (PerkinElmer, Waltham, MA, USA). Antibodies for Ghrelin, PYY_3-36_ and GLP-1 were purchased from Bachem (T-4747, T-4090 and T-4056, respectively), while CCK-8 antibody C2581 was purchased from Sigma Aldrich (St. Louis, MO, USA). Detection limits and coefficient of variations (CV) for each of the RIA measured hormones are as follows: Ghrelin: 50–3200 pg/mL, inter-assay CV 13%, intra-assay CV 10%; GLP-1: 7.5–1000 pg/mL, inter-assay CV 12%, intra-assay CV 9%; PYY_3-36_: 3.7–250 pg/mL, inter-assay CV 14%, intra-assay CV 8%; CCK-8: 0.62–80 pg/mL, inter-assay CV 13%, intra-assay CV 8%.

Breath samples were analyzed using the Quintron GaSampler System (Qunitron Instruments) for hydrogen content.

### 2.6. Statistical Analysis

A power calculation indicated that a sample of 43 participants would be sufficient to detect a 150 kcal difference in food intake at *p* < 0.05 and beta = 0.9. Forty-eight participants were recruited to allow for attrition. Means and standard error were calculated for all study variables. Treatment effects of SFD on subjective appetite measures, hormone response and breath hydrogen data were analyzed with a mixed-model ANCOVA (analysis of covariance) using treatment and time point as repeated measures and baseline as a covariate. Treatment effects on food intake were analyzed using a one-way, repeated measures analysis of variance (ANOVA). All post-hoc, pairwise comparisons were performed using Bonferroni adjustments. Statistical analysis was conducted using SPSS for Windows or Mac (version 16.0; SPSS, Chicago, IL, USA).

## 3. Results

### 3.1. Participant Demographics

In total, 68 people completed the screening process for this study. Twenty were found to be ineligible with the two most common disqualifying factors being BMI outside of the target range (6 people) and low palatability ratings for the test foods (12 total: 2 for test beverage, 9 for test bar, 1 for pasta lunch). Forty-eight participants were randomized into the study. One male participant dropped out after completing two sessions due to an unrelated health issue. Four participants (3 female, 1 male) dropped out after four sessions due to schedule conflicts. Forty-three participants completed the study (22 male and 21 female). Average age was 25 ± 3.6 years with a BMI of 23.9 ± 2.9 kg/m^2^.

### 3.2. Breath Hydrogen

**Figure 1:**
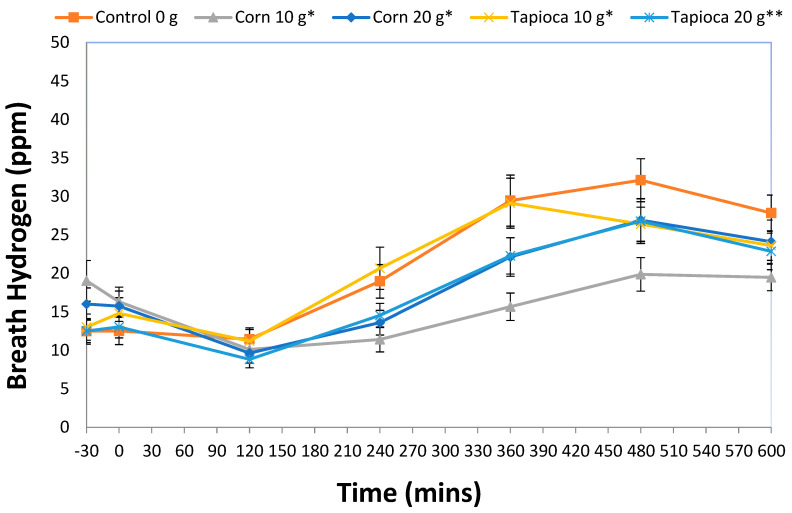
Mean ± standard error (SE) for breath hydrogen rated through 600 min. * Indicates statistical significance from control (*p* < 0.05). ** Indicates statistical significance from both Corn10 and Tapioca10 treatments (*p* < 0.05).

Figure 1 shows mean breath hydrogen results by treatment. The mixed-model ANCOVA revealed a significant overall treatment effect (*F*(4,527) = 17.0, *p* < 0.001) with all SFD treatments having significantly higher breath hydrogen than control (*p* < 0.05). Post-hoc analysis showed a dose-response for tapioca-based SFD with Tapioca20 having significantly higher breath hydrogen than both Tapioca10 and Corn10 (*p* < 0.001 for both). Corn-based SFD showed a trend towards a dose-response, with Corn20 having slightly higher breath hydrogen response when compared to Corn10 and Tapioca10 (*p* = 0.066 and 0.052, respectively).

### 3.3. Hormones

**Figure 2:**
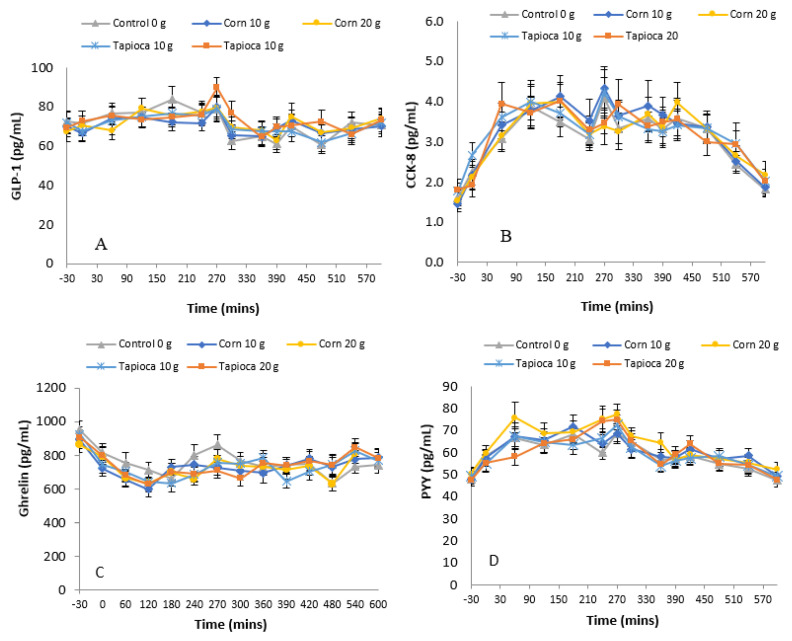
Plasma hormone responses for GLP-1 (**A**), cholecystokinin (**B**), ghrelin (**C**), and peptide YY (**D**) measured from baseline through 600 min. Indicates statistical significance from control (*p* < 0.05). GLP-1: Glucagon-Like-Peptide-1, CCK-8: Cholecystokinin, PYY_3-36_: Petptide YY.

Figure 2 shows data for the plasma concentration of GLP-1, ghrelin, CCK-8 and PYY_3-36_. Statistical analysis did not reveal an overall treatment difference for any hormone measures: GLP-1 (*F*(4,683) = 1.478; *p* > 0.05), ghrelin (*F*(4,571) = 0.372; *p* > 0.05), CCK-8 (*F*(4,659) = 0.851; *p* > 0.05) and PYY_3-36_ (*F*(4,659) = 2.262; *p* > 0.05). Post-hoc analysis, however, showed significantly higher PYY_3-36_ concentrations for the Corn20 treatment as compared to control (*p* = 0.043).

### 3.4. Subjective Appetite

**Figure 3:**
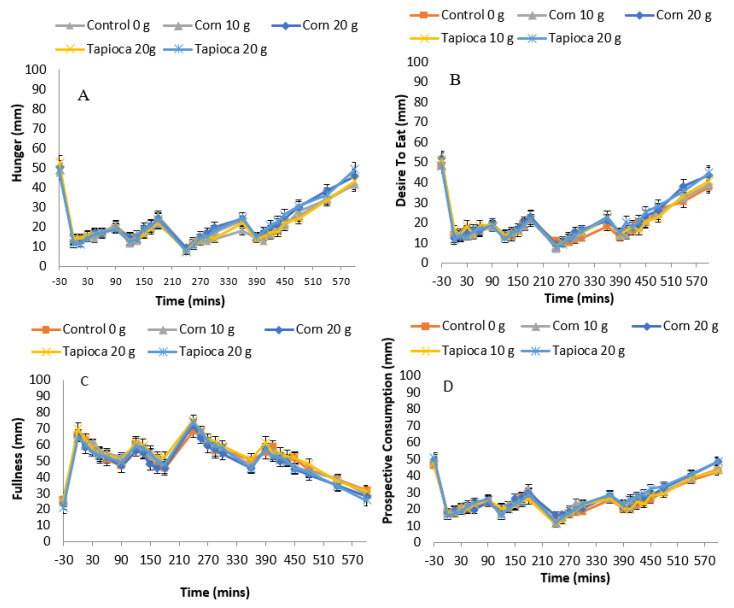
VAS (visual analogue scale) scores of hunger (**A**), fullness (**B**), desire to eat (**C**) and prospective consumption (**D**) rated from baseline through 600 min following control (triangles), Corn10 (diamonds), Corn20 (circles), Tapioca10 (star) and Tapioca20 (square) treatments. There were no statistically significant treatment differences observed (*p* > 0.05).

Figure 3 shows mean appetite ratings for hunger, fullness, desire to eat and prospective consumption from baseline through 10 h (600 min) post-consumption of the treatment beverage. Repeated measures ANCOVA showed no significant main treatment effects on hunger (*F*(4,603) = 2.722; *p* > 0.05), fullness (*F*(4,853) = 1.720; *p* > 0.05), desire to eat (*F*(4,679) = 0.909; *p* > 0.05) and prospective consumption (*F*(4,766) = 1.247; *p* > 0.05).

### 3.5. Food Intake

Food intake measures consisted of in-laboratory ad libitum lunch, afternoon snack and dinner intake, as well as evening food intake measured from participant diet diaries. Table 1 shows mean intakes for each treatment. Statistical analysis revealed no main treatment effect on calories consumed from the lunch, snack, evening meal or consumption over the rest of the day (diet diary intake). Additionally, when the breakfast and mid-morning snack were factored in and total caloric intake for the test day was analyzed, there were no treatment differences observed.

## 4. Discussion

This present study investigated the effect of consuming SFD on food intake, subjective appetite, plasma concentration of several satiety-related hormones and breath hydrogen (an indirect marker of colonic fermentation of carbohydrates). Our primary hypothesis was that consuming SFD would reduce food intake. However, regardless of the dose or source, SFD did not reduce food intake. In addition, consuming SFD had no statistically significant effect on subjective appetite, or plasma hormone concentration. However, consuming SFD did increase breath hydrogen.

The results obtained by this present study do not support previous studies that have found that consuming SFD reduces food intake or subjective appetite [13,15]. There are several possible explanations for these discrepant results, including differences in the study group or experimental design. However, a significant difference between studies is the vehicle used to deliver the SFD. Previous studies have used a beverage as the vehicle for delivering SFD, whereas in this present study, a beverage and a solid food were used. Little is currently known about the effect of fiber in beverages on food intake and appetite. A number of studies have found that beverages are less satiating than solid foods and may not elicit a robust satiety response [24,25,26]. A reduced satiety response holds regardless of the macronutrient content of the beverage [27]. However, previous research using SFD reported an effect on food intake despite being consumed in a beverage [9]. It is not clear why we found different results, but it could be due to differences in the vehicle, study group, the nature of the breakfast meal or the time of day the SFD was administered. It is possible that dietary fiber influences satiety through different mechanisms than the other macronutrients, rendering the form in which it is consumed to be less important. Further studies are required to elucidate the effect of fiber in beverages on satiety.

Another possibility is that the solid food used in this present study had a strong satiating effect independent of the resistant dextrin content, which acted to mask an effect of SFD on appetite. In addition to vehicle influences, participants were required to spend the entire day in the laboratory and the change in their activity levels or eating patterns may have contributed to an attenuated appetite response [28,29]. Further research is warranted to understand how the form in which SFD is consumed influences appetite and to determine the optimum vehicle for its delivery.

We hypothesized that SFD would be fermented in the colon. Indeed, breath hydrogen was higher following consumption of SFD, providing indirect evidence that SFD was fermented. A dose-response relationship was observed but there was no statistically significant difference between the sources of SFD (tapioca or corn). SFD appears to be readily fermented and the peak breath hydrogen concentration was higher following consumption of the SFD compared to recent studies of gel-forming pectin [30] or fructo-oligosaccharides [31]. However, we failed to observe any statistically significant correlation between breath hydrogen and any questionnaire responses or biochemical markers of satiety (data not shown). This finding is in agreement with a recent study that found no relationship between breath hydrogen and subjective appetite despite a statistically significant correlation between breath hydrogen and GLP-1 being observed [32].

Previous studies found that the plasma concentration of GLP-1 and PYY_3-36_ were increased when rodents ate a diet enriched with resistant starch [18]. By contrast, plasma concentration of GLP-1 was reduced in a human study following the consumption of a test food containing resistant starch [33]. In addition, cell studies have shown that SCFA stimulate the release of PYY_3-36_ and GLP-1 from colonic cells [17,34,35]. This present study found no effect of consuming SFD on plasma concentration of CCK, ghrelin, PYY_3-36_ or GLP-1. These discrepant results may be due to differences in the type of fiber used and the dose provided, differences in the study group or differences in the experimental design. Further research is warranted to confirm that these biomarkers of appetite respond to the consumption of SFD through increased SCFA production, resulting from colonic fermentation. Moreover, the dose of SFD required to robustly elicit an effect is required.

This present study recruited approximately equal numbers of male and female participants. All data are reported for the total study group and they were not stratified by sex or gender. This may mask important differences between the male and female participants. For instance, the males and females may have responded to the intervention, albeit in opposite directions, thus cancelling each other out when their responses were combined into one study group. This study was not designed or powered to determine sex differences and no a priori hypothesis was made regarding gender or sex effect. Consequently, conducting post hoc subset analysis may provide misleading results [36]. However, in the preliminary analysis, we saw no evidence of sex or gender differences in any of the outcome measures. Recent studies report gender differences in the biological response to meal ingestion [37], or appetitive responses and food intake [38], due to the phase of the menstrual cycle. We did not determine the menstrual phase of the female participants in this study which also may have masked an effect on our outcome measures. Future studies that are powered to identify sex or gender differences, that use carefully developed inclusion/exclusion criteria that are chosen with thought to their sex and gender impact, and control for the phase of the menstrual cycle, should be conducted to robustly determine sex or gender effects of this intervention.

This present study has a number of limitations that must be considered when interpreting the data. The participants’ food choices and meal-times were dictated by the research team. Consequently, this may have interfered with the normal expression of appetite and food intake. Moreover, free food was supplied which may have stimulated overconsumption, thereby masking an effect of SFD on food intake [39]. These issues are common to laboratory-based studies where external validity is low [40,41]. It is possible that a study of free-living individuals may highlight other effects of SFD on appetite (e.g., extending time between meals, reduced snacking and reduced intake at self-selected meal-times). This study was also a single exposure study that sought to understand an effect on food intake and the biological underpinnings of any observed effect. Further studies would need to be conducted to determine the long-term effect of consuming SFD on food intake and body weight. The study group had relatively narrow characteristics and it should not be assumed that these results will hold in other groups (e.g., obese individuals, children, or older adults). Further studies are required to understand how different groups respond to the ingestion of SFD. Moreover, due to logistical reasons, we did not control for the menstrual cycle in the female participants. The limitations of this study may have individually or collectively masked an effect of SFD on food intake and appetite. Studies using different approaches are required to fully understand the effect of SFD on food intake and appetite.

## Figures and Tables

**Table 1:** Food intake data.

Treatment	Lunch	Afternoon Snack	Dinner	Food Logs	Total (Including Breakfast and Morning Snack)
Control	441 ± 33	263 ± 18	499 ± 38	290 ± 44	2342 ± 90
Corn10	452 ± 33	282 ± 20	534 ± 38	358 ± 60	2489 ± 94
Corn20	457 ± 27	268 ± 19	511 ± 36	337 ± 60	2440 ± 85
Tapioca10	444 ± 31	264 ± 18	481 ± 29	360 ± 51	2412 ± 84
Tapioca20	422 ± 30	239 ± 18	502 ± 36	276 ± 48	2316 ± 95
*F*(4,39)	0.808	1.583	0.798	0.741	2.040
Main Effect *p*-Value	0.528	0.198	0.534	0.570	0.108

Means (kcal) ± SE.
